# Mi2β Is Required for γ-Globin Gene Silencing: Temporal Assembly of a GATA-1-FOG-1-Mi2 Repressor Complex in β-YAC Transgenic Mice

**DOI:** 10.1371/journal.pgen.1003155

**Published:** 2012-12-20

**Authors:** Flávia C. Costa, Halyna Fedosyuk, Allen M. Chazelle, Renee Y. Neades, Kenneth R. Peterson

**Affiliations:** 1 Department of Biochemistry and Molecular Biology, University of Kansas Medical Center, Kansas City, Kansas, United States of America; 2 Department of Anatomy and Cell Biology, University of Kansas Medical Center, Kansas City, Kansas, United States of America; Stanford University School of Medicine, United States of America

## Abstract

Activation of γ-globin gene expression in adults is known to be therapeutic for sickle cell disease. Thus, it follows that the converse, alleviation of repression, would be equally effective, since the net result would be the same: an increase in fetal hemoglobin. A GATA-1-FOG-1-Mi2 repressor complex was recently demonstrated to be recruited to the −566 GATA motif of the ^A^γ-globin gene. We show that Mi2β is essential for γ-globin gene silencing using Mi2β conditional knockout β-YAC transgenic mice. In addition, increased expression of ^A^γ-globin was detected in adult blood from β-YAC transgenic mice containing a T>G HPFH point mutation at the −566 GATA silencer site. ChIP experiments demonstrated that GATA-1 is recruited to this silencer at day E16, followed by recruitment of FOG-1 and Mi2 at day E17 in wild-type β-YAC transgenic mice. Recruitment of the GATA-1–mediated repressor complex was disrupted by the −566 HPFH mutation at developmental stages when it normally binds. Our data suggest that a temporal repression mechanism is operative in the silencing of γ-globin gene expression and that either a trans-acting Mi2β knockout deletion mutation or the *cis*-acting −566 ^A^γ-globin HPFH point mutation disrupts establishment of repression, resulting in continued γ-globin gene transcription during adult definitive erythropoiesis.

## Author Summary

Sickle cell disease (SCD) is one of the most common genetic diseases, affecting millions of people worldwide. SCD affects red blood cells' shape and renders them ineffective, resulting in anemia along with attendant complications. The disease is caused by a single point mutation in the coding sequence of the adult β-globin gene that changes normal adult hemoglobin (HbA) to sickle hemoglobin (HbS). Scientific evidence has demonstrated that continued expression of the fetal γ-globin genes (fetal hemoglobin, HbF), which are normally silenced after birth, is the best treatment for SCD, since the pathophysiology is largely ameliorated. Our therapeutic goal is to reactivate the γ-globin genes to substitute for the defective adult β-globin gene. We identified a novel γ-globin gene silencer sequence and demonstrated that a GATA-1-FOG-1-Mi2 repressor complex binds to this sequence and silences γ-globin synthesis. However, data regarding the requirement of Mi2 for silencing is controversial. We demonstrate that γ-globin synthesis increases as Mi2 expression decreases. We also show that repressor complex components assemble sequentially during development; completion of assembly coincides with γ-globin gene silencing. Disruption of either the repressor complex or mutation of its binding site induces γ-globin. Understanding this mechanism will reveal potential new targets for treating SCD.

## Introduction

The human β-globin locus is composed of five functional genes (ε, ^G^γ, ^A^γ, δ, and β) and a master regulatory region called the locus control region (LCR). These genes are arrayed in the order in which they are progressively expressed during development. Expression of the β-like globin genes undergoes two major switches. The first is an embryonic to fetal switch that occurs between 6 and 8 weeks of gestation and involves the silencing of the embryonic ε-globin gene in the yolk sac and the activation of the fetal γ-globin genes (^A^γ- and ^G^γ-globin) in the liver. The second switch is from the fetal γ-globins in the liver to the adult globins (mostly β-globin, with δ-globin as a minor component) in the bone marrow. This switch is characterized by the progressive silencing of the γ-globin genes, with the concomitant activation of β-globin gene expression, and is not completed until after birth. An understanding of the mechanisms that regulate the globin gene switching is of fundamental importance, since reactivation of the fetal hemoglobin expression during definitive erythropoiesis is well-established as therapeutic for hemoglobinopathies such as sickle cell disease (SCD) and β-thalassemias.

Hereditary persistence of fetal hemoglobin (HPFH) is a condition characterized by elevated synthesis of γ-globin in adult definitive erythroid cells, which normally have only very low levels of fetal hemoglobin (HbF). HPFH mutations include both small and large deletions in the β-globin locus (deletional HPFH), as well as point mutations in the two γ-globin gene promoters (non-deletional HPFH). When a HFPH mutation is co-inherited with a SCD mutation, the SCD patients present with a better clinical evaluation due to the high levels of HbF.

We identified a novel ^A^γ-globin gene silencer motif and an associated repressor complex that are linked to a new HPFH point mutation [1]. This silencer is located at −566 relative to the mRNA CAP site in a GATA binding motif and repression is mediated by GATA-1 binding at this site, with Friend of GATA-1 (FOG-1) and Mi2 (NuRD) as protein partners in this repressor complex. Interestingly, a mutation in the analogous −567 GATA site of the ^G^γ-globin gene in an Iranian-American family was recently associated with a HPFH phenotype and GATA-1 protein was shown to bind at this site when γ-globin is not expressed [2]. Together, these studies demonstrate that the −566 ^A^γ- and −567 ^G^γ-globin GATA sites are true silencers and that the GATA-1 protein is the DNA-binding component that mediates γ-globin gene silencing.

GATA-1 is a zinc finger transcription factor that plays a role during development in the differentiation of several cell types including erythrocytes, megakaryocytes, eosinophils and mast cells [3]. GATA-1 recognizes the consensus sequence (A/T) GATA (A/G) and, like many other transcription factors, binds to its cognate DNA sequence, facilitating target gene repression or activation through recruitment of co-activator or co-repressor proteins [4]. Previously published studies demonstrate that GATA-1 is capable of acting both as an activator and a repressor of transcription [1], [5], [6]. GATA-1 binds the co-regulator FOG-1, which assists in potentiating transcriptional activation or repression [7], [8]. These two proteins were shown to associate with the NuRD complex and mediate the repression of certain genes, including γ-globin [1], [5]. A repressive GATA-1/FOG-1/MeCP1 complex binds to silenced hematopoietic genes in erythroid cells, with FOG-1 serving as the bridging factor between GATA-1 and the MeCP1 complex [6]. A recent study demonstrated that the GATA-1/FOG-1/NuRD complex is also associated with gene activation [9], [10].

In this study, we demonstrate that Mi2β is required for γ-globin gene silencing. γ-globin was increased in definitive erythroid cells from Mi2β conditional knockout human β-globin locus yeast artificial chromosome (β-YAC) transgenic lines, corroborating the involvement of Mi2 (NuRD) in establishing the permanent silencing γ-globin gene expression. In addition, we focused on the temporal events leading to GATA-1-FOG-1-Mi2-mediated γ-globin gene silencing. We hypothesized that repression is established gradually over time in the developing mouse fetus. Chromatin immunoprecipitation (ChIP) experiments performed on post-conception day E12–E18 fetal liver samples from β-YAC transgenic mice showed that GATA-2 occupies the −566 ^A^γ-globin GATA site early in fetal liver definitive erythropoiesis when γ-globin is expressed (day E12). GATA-2 vacates this site and is replaced by GATA-1 at day E16, followed by recruitment of FOG-1 and Mi2 proteins at day E17. Finally, we demonstrate that γ-globin is expressed during adult definitive erythropoiesis in β-YAC transgenic mice carrying the T>G HPFH point mutation at the −566 GATA motif of the ^A^γ-globin gene. The presence of this mutation disrupted recruitment of the GATA-1-FOG-1-Mi2 repressor complex to this motif, resulting in reactivation of γ-globin expression during adult definitive erythropoiesis.

## Results

### Expression of γ-globin in Mi2β conditional knockout β-YAC mice

**Figure 1:**
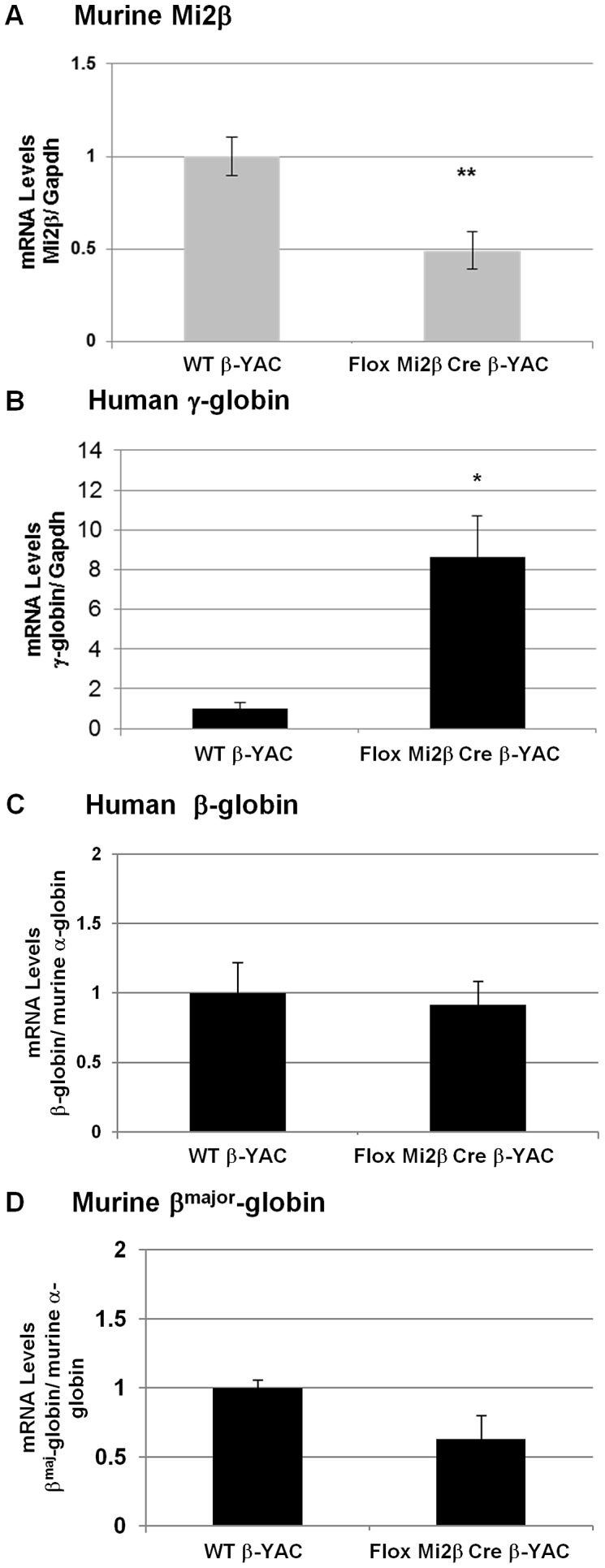
Expression of murine Mi2β and human and murine β-like globins in erythroid-specific conditional knockout Mi2β β-YAC transgenic mice. Expression of murine Mi2β, human fetal γ-globin, human adult β-globin and murine adult β^maj^-globin was analyzed by real-time qRT-PCR in adult blood samples. A) Murine Mi2β; B) Human γ-globin; C) Human β-globin; D) Murine β^maj^-globin. Numbers represent the average of 6 animals (* indicates P<0.05).

The NuRD complex is composed of the ATPase Mi2, MTA-1, MTA-2, p66, RbAp46 (RBBP7), RbAp48 (RBBP4), MBD3 and the histone deacetylases HDAC1 and HDAC2 [11]. Given the association of NuRD with other transcriptional repressors and the presence of a histone deacetylase and an ATPase subunit in this remodeling complex, NuRD is frequently associated with transcriptional repression [11], [12]. Earlier ChIP experiments demonstrated that Mi2 is recruited to the −566 GATA site of the ^A^γ-globin gene when γ-globin is no longer expressed [1]. To further examine the role of Mi2 in the silencing of γ-globin expression, a conditional knockout of Mi2β was created by breeding floxed Mi2β mice [13] with our erythroid-specific Cre expression mice [14] and our wild-type β-YAC transgenic mice [15] as described in Materials and Methods. Six mice were obtained and correct genotypes were determined. Conditional knockout of the murine Mi2β gene in our mice was demonstrated at the transcript level by real-time qRT-PCR. Mi2β mRNA expression was reduced to 50% (average of 6 animals, P<0.01) in peripheral blood samples from these mice compared to wild-type β-YAC mice (Figure 1A). Expression of the murine globins (ε^y^, βh1, and β^maj^) and human globins (γ and β) was analyzed by qRT-PCR and compared to wild-type β-YAC transgenic mice. Human γ-globin gene expression was increased 8-fold in peripheral blood from adult conditional Mi2β knock-out mice (P<0.05, Figure 1B). Human β-globin and murine adult β^maj^-globin gene expression were decreased, but not significantly (Figure 1C–1D). The murine embryonic βh1- and ε^y^-globins were expressed at the same level as in wild-type β-YAC mice (data not shown).

**Figure 2:**
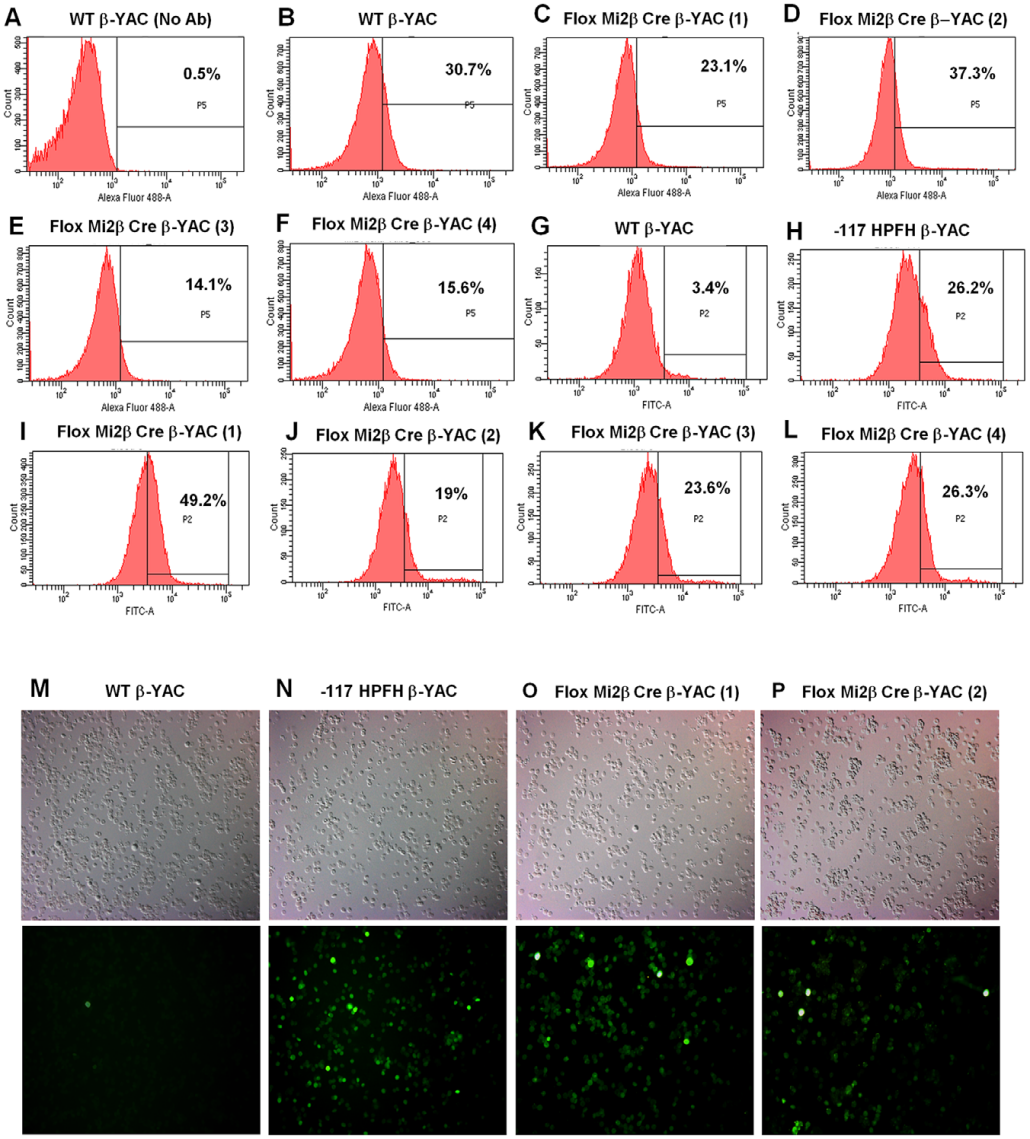
Flow cytometry and cytospin analysis of blood from adult erythroid-specific Mi2β conditional knockout β-YAC transgenic mice. A rabbit polyclonal anti-Mi2 antibody+secondary antibody (panels A–F) or an anti-human hemoglobin F FITC-conjugated antibody (panels G–L) was used to determine the percentage of cells expressing of Mi2 or HbF, respectively. Cytospins were prepared using an anti-human hemoglobin F FITC-conjugated antibody (panels M–P). Antibody employed, followed by sample: A) IgG control, wild-type β-YAC; B) Mi2, wild-type β-YAC; C–F) Mi2, floxed Mi2β μ'LCR β pr-Cre β-YAC 1, 2, 3 and 4, respectively; G) HbF, wild-type β-YAC; H) HbF, −117 Greek HPFH β-YAC; I–L) Mi2 floxed Mi2β μ'LCR β pr-Cre β-YAC 1, 2, 3 and 4, respectively. Cytospins: M) wild-type β-YAC; N) −117 Greek HPFH β-YAC; O–P) floxed Mi2β μ'LCR β pr-Cre β-YAC 1 and 2. Top panels, visible light images of cytospin cells; bottom panels, immunofluorescent images of same fields.

Expression at the protein level confirmed the transcription results. Mi2 protein expression was decreased nearly 50% in adult blood from two of the Mi2β conditional knockout mice (3 and 4) mice (Figure 2E and 2F) compared to wild-type mice (Figure 2B) as measured by flow cytometry. The other two Mi2β conditional knockout mice (1 and 2) showed a modest 20% decrease or no decrease in Mi2 protein expression, respectively (Figure 2C and 2D). Taken together these data indicate variability of Cre excision efficiency among the mice. γ-globin (HbF)-expressing F cells were measured in parallel (Figure 2G–2L). Although all Mi2β knockout mice showed substantial increases in F cells, levels were variable and not concordant with the decrease in Mi2β expression. Additionally, cystospins of adult peripheral blood from two Mi2β conditional knockout mice queried with anti-human HbF antibody displayed a pancellular distribution of F cells (Figure 2O–2P), similar to the −117 Greek HPFH β-YAC mice (Figure 2N), although the Mi2β conditional knockout mice showed fewer strong HbF-positive cells.

**Figure 3:**
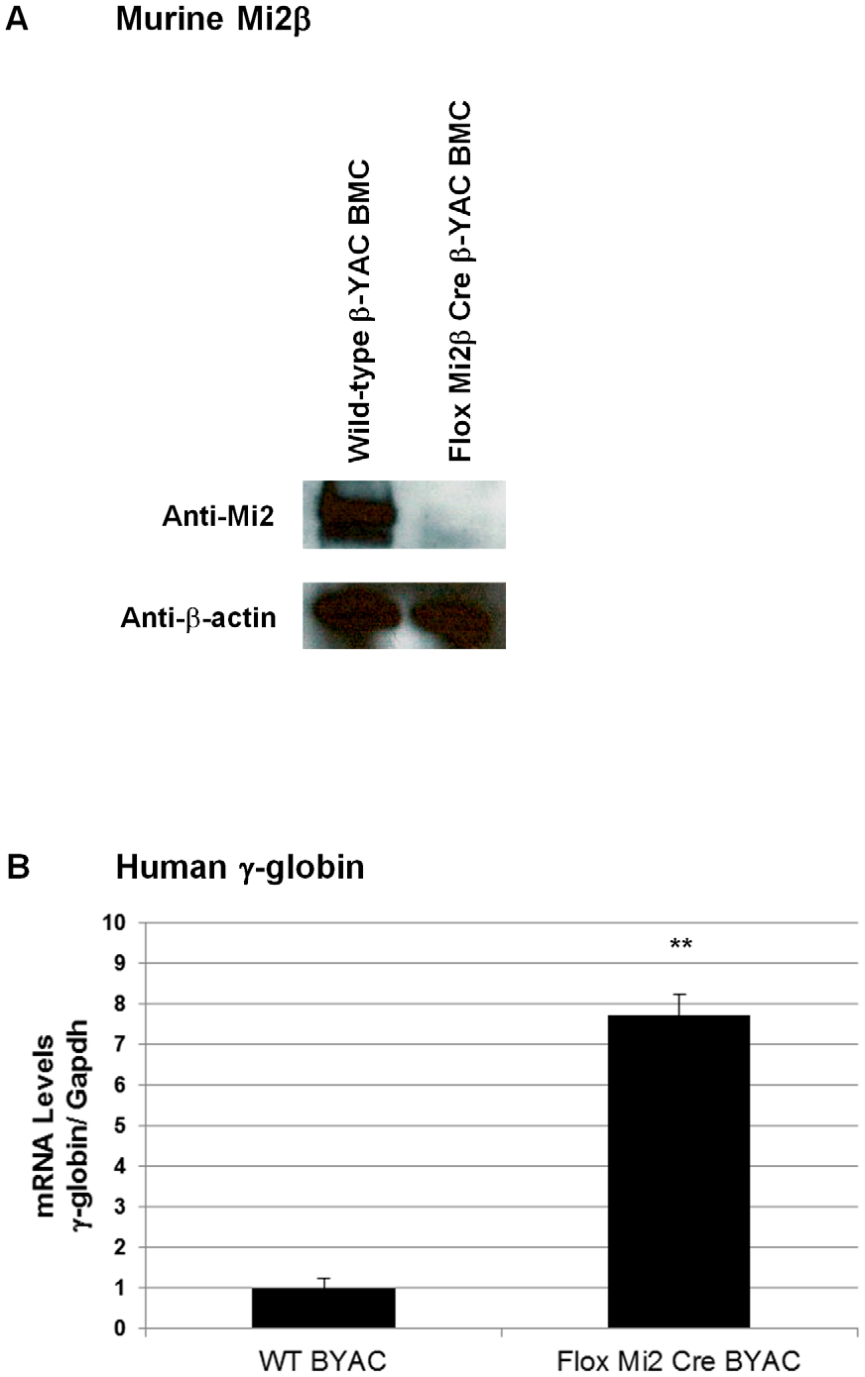
Murine Mi2β protein and human fetal γ-globin mRNA expression in CID-dependent bone marrow cells (BMCs) derived from Mi2β conditional knockout β-YAC transgenic mice. mRNA and protein levels from in CID-dependent wild-type β-YAC and floxed Mi2β μ'LCR β pr-Cre β-YAC BMCs were analyzed by Western blotting and real-time qRT-PCR, respectively. A) Mi2 Western blotting using an anti-Mi2 antibody; B) γ-globin mRNA.

Mature RBCs are enucleated, making it difficult to demonstrate that the nuclear-localized Mi2β protein is reduced in these cells. To further demonstrate decreased expression of Mi2β protein in our conditional knockout mice, we derived nucleated CID-dependent bone marrow cells (BMCs) from our Mi2β conditional knockout mice. BMCs obtained and immortalized in this manner reflect the globin gene expression pattern observed in the adult transgenic mice from which they are derived [16]. Western blotting was performed using an anti-Mi2β antibody; a 240 KDa fragment corresponding to Mi2β was detected in CID-dependent wild-type β-YAC BMCs, but not in the CID-dependent Mi2β conditional knockout β-YAC BMCs (Figure 3A). Real time PCR corroborated this result at the transcript level (data not shown). Finally, a 7.5-fold induction of γ-globin mRNA level was measured in the CID-dependent Mi2β conditional knockout β-YAC BMCs relative to CID-dependent wild-type β-YAC BMCs (Figure 3B). Together, these data confirm the role of Mi2 as an essential component of the γ-globin silencing complex.

### Temporal repression of γ-globin by sequential recruitment of GATA-1, FOG-1, and Mi2 to the ^A^γ-globin −566 GATA silencer

**Figure 4:**
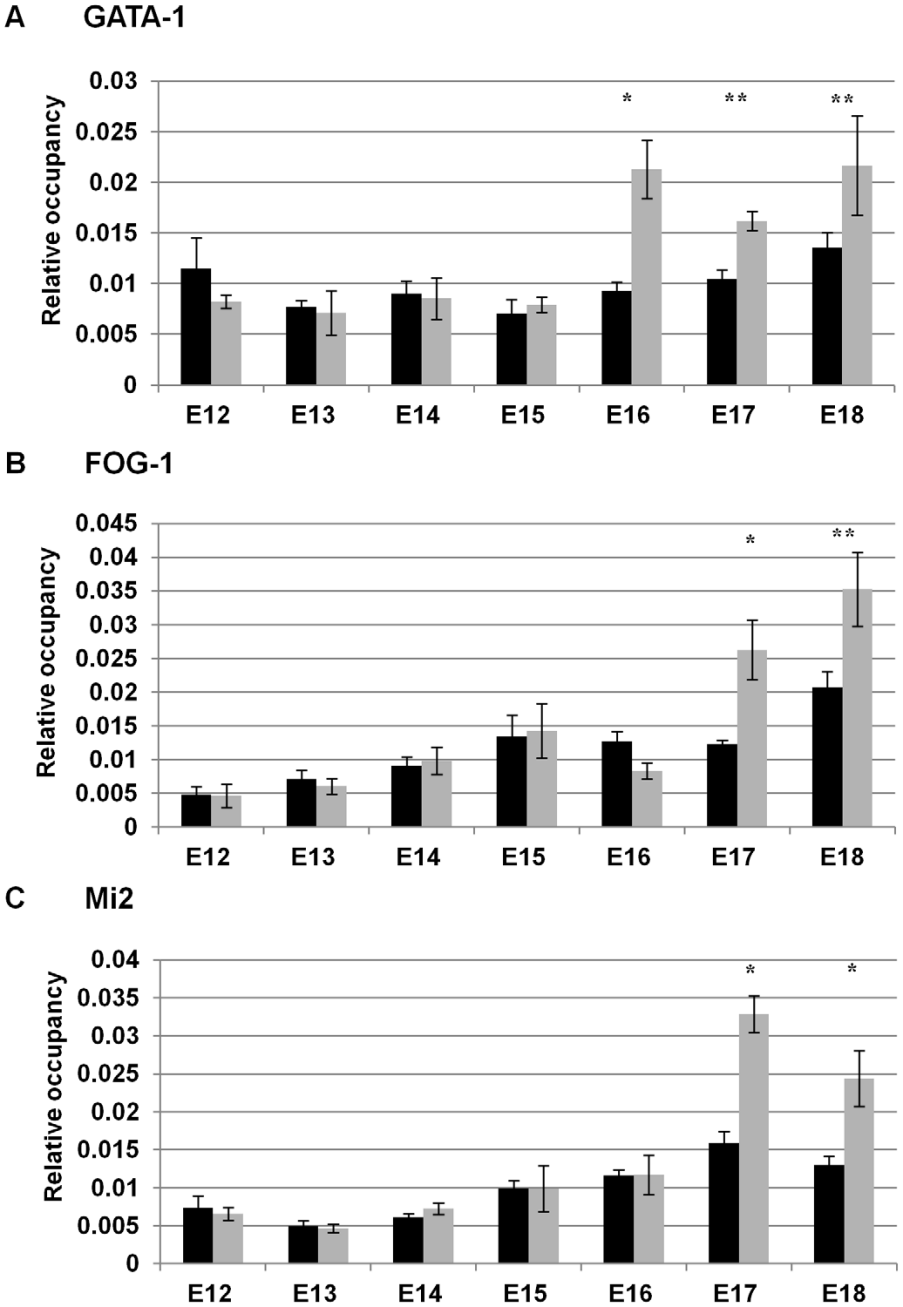
Temporal recruitment of the GATA-1-FOG1-Mi2 complex to the −566 ^A^γ-globin GATA motif in wild-type β-YAC. ChIP analysis of the −566 ^A^γ-globin GATA site in fetal liver samples from post-conception days E12–E18 wild-type β-YAC transgenic conceptuses. The relative occupancy of the −566 region of the ^A^γ-globin gene (GenBank coordinates 38772–38937 from accession file GI455025) by GATA-1, FOG-1, and Mi2 proteins (grey bars) is shown in comparison to IgG control samples (black bars). ChIP was carried out using isotype-matched immunoglobulin (IgG), GATA-1, FOG-1 and Mi2 antibodies (Santa Cruz Biotechnology, Santa Cruz, CA) as described in Materials and Methods. The results are the average of at least two experiments and each experiment was performed in duplicate (* indicates P<0.05 and ** P<0.01). A) GATA-1 protein occupancy; B) FOG-1 protein occupancy; C) Mi2 protein occupancy.

We previously demonstrated that GATA-1 was recruited to the ^A^γ-globin −566 GATA silencer by day E18 in fetal liver from wild-type β-YAC transgenic mice, a developmental time point at which γ-globin is no longer expressed [1]. GATA-1 was not present at this site at day E12, when γ-globin is at its highest expression level in the fetal liver. However, we did not examine recruitment during the intervening days. Thus, the assembly of the GATA-1 repressor complex at the −566 silencer region might occur in a sequential manner, with each component recruited in a temporal fashion between days E12 and E18 with GATA-1 recruitment coinciding with the onset of γ-globin gene silencing. To test this hypothesis, chromatin immunoprecipitation (ChIP) analyses were performed using wild-type β-YAC transgenic mouse staged fetal liver samples from days E12 to E18. Our data demonstrated that GATA-1, FOG-1 and Mi2 proteins do not occupy the −566 GATA silencer until day E16 and E17 (Figure 4A–4C). Although no recruitment was demonstrated until day E16 when silencing begins, we observed a temporal recruitment of the previously identified repressor components. GATA-1 alone occupied the −566 GATA silencer at day E16 (Figure 4A), but FOG-1 or Mi2 occupancy was not observed until day E17 (Figure 4B and 4C, respectively). The complete GATA-1/FOG-1/Mi2 protein complex was observed at day E18 as previously demonstrated [1].

### GATA-2 occupies the −566 ^A^γ-globin silencer prior to repression by GATA-1/FOG-1/Mi2

**Figure 5:**
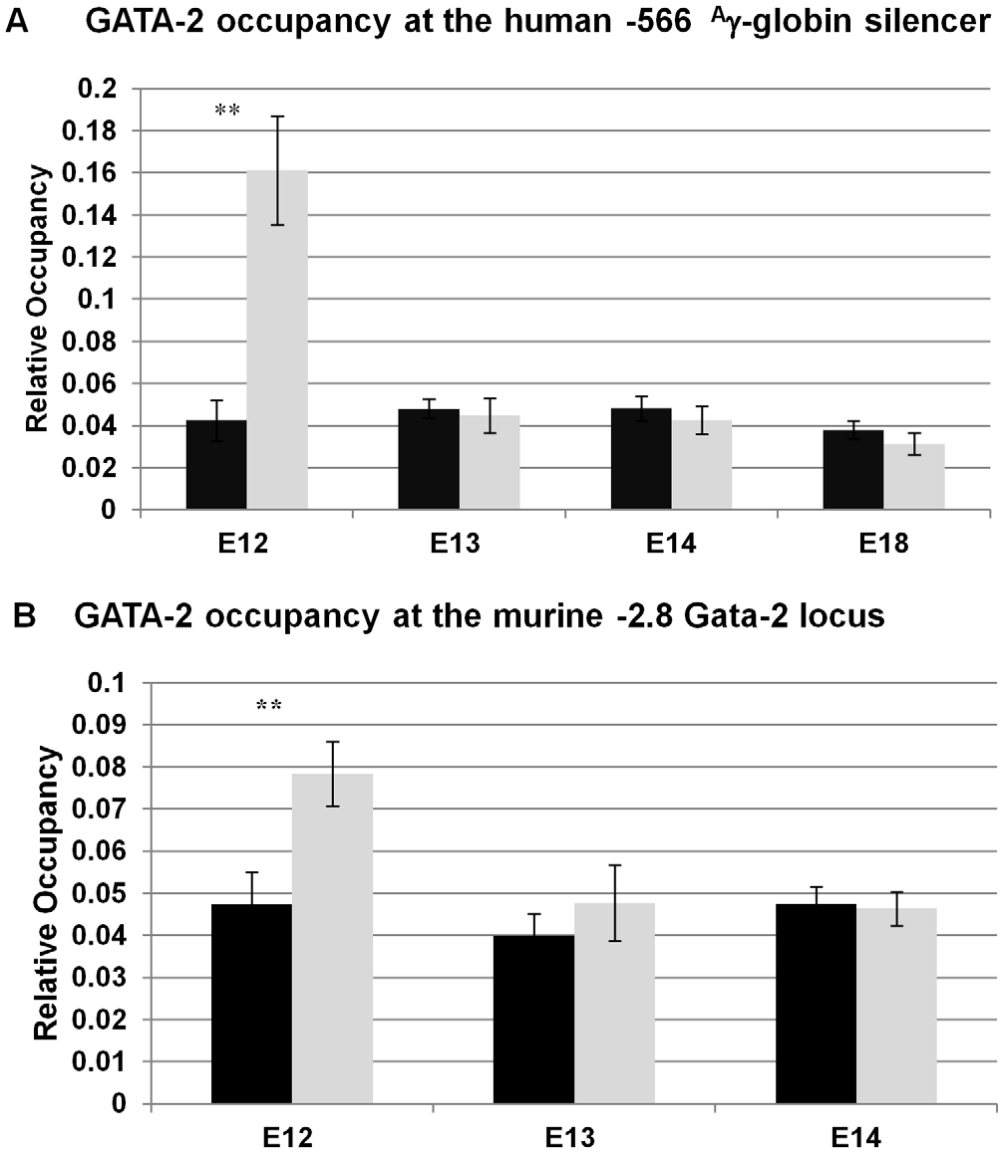
GATA-2 binding at the −566 GATA site of the ^A^γ-globin gene in wild-type β-YAC. A) GATA-2 protein occupancy at the −566 ^A^γ-globin GATA site by ChIP analysis. B) GATA-2 protein occupancy at the murine Gata-2 gene −2.8 Kb binding site by ChIP analysis. Binding of GATA-2 at the murine Gata-2 locus was used as a positive control for GATA-2 occupancy. ChIP was performed as described in the legend to Figure 4; labeling is also as illustrated in Figure 4.

GATA-1 and GATA-2 are reciprocally expressed during erythropoiesis, with GATA-1 levels rising when GATA-2 levels decline [17], [18]. GATA-1 and GATA-2 share a common WGATAR DNA motif, present at *cis*-regulatory elements that activate transcription in an erythroid cell-specific manner [18]. These data prompted us to investigate whether GATA-2 was bound to the −566 GATA silencer prior to GATA-1-mediated repression, even though GATA-2 is thought to not play a role in globin gene switching once the erythroid lineage has been established [3]. ChIP experiments were performed using day E12 and E18 fetal liver samples from wild-type β-YAC mice, where we previously demonstrated the absence (day E12) and presence (day E18) of GATA-1 recruitment. GATA-2 occupancy was observed in day E12 samples from the wild-type β-YAC transgenic mice (Figure 5A). Occupancy at the Gata-2–2.8 Kb region by GATA-2, a positive control, was observed in day E12 samples (Figure 5B), but not in day E18 samples from these mice (data not shown). This control is consistent with previous data where GATA-2 was demonstrated to bind the −2.8 Kb region of the Gata-2 locus when the locus is transcriptionally active, but is replaced by GATA-1 to initiate repression [19], [20].

Taken together, our results support a model of temporal repression, in which GATA-2 first occupies the −566 ^A^γ-globin silencer at day E12, followed by GATA-1 occupancy at day E16 and FOG-1 and Mi2 at day E17. The γ-globin silencing might be initiated by the change in the GATA factor occupancy at the −566 GATA motif, suggesting that GATA switches may play a role as a determinant of the onset of temporal repression by GATA-1 at the −566 silencer region.

### Generation of −566 ^A^γ-globin HPFH β-YAC transgenic lines

To definitively prove that a HPFH mutation identified by us and another group [1], [2] had the expected phenotype, we introduced the T>G mutation at position −566 relative to the ^A^γ-globin mRNA start site into the normally located copy of the ^A^γ-globin gene in the β-YAC and produced transgenic mice. The GATA to GAGA alteration (and the absence of others) was confirmed by DNA sequence analysis of a PCR product amplified from the promoter region of the resultant YAC. Three −566 T>G ^A^γ-globin HPFH β-YAC transgenic lines were obtained (lines 18, 20 and 25). Structural analysis was performed using radioactively-labeled DNA probes spanning the locus from 5′HS3 through the HPFH6 breakpoint on Southern blots of pulsed-field gels to confirm integrity of the β-globin transgene loci and copy numbers were determined as described in Materials and Methods (data not shown). Only line 20 was suitable for further analysis.

### The −566 T>G ^A^γ-globin HPFH mutation maintains γ-globin expression in adult definitive erythropoiesis

**Figure 6:**
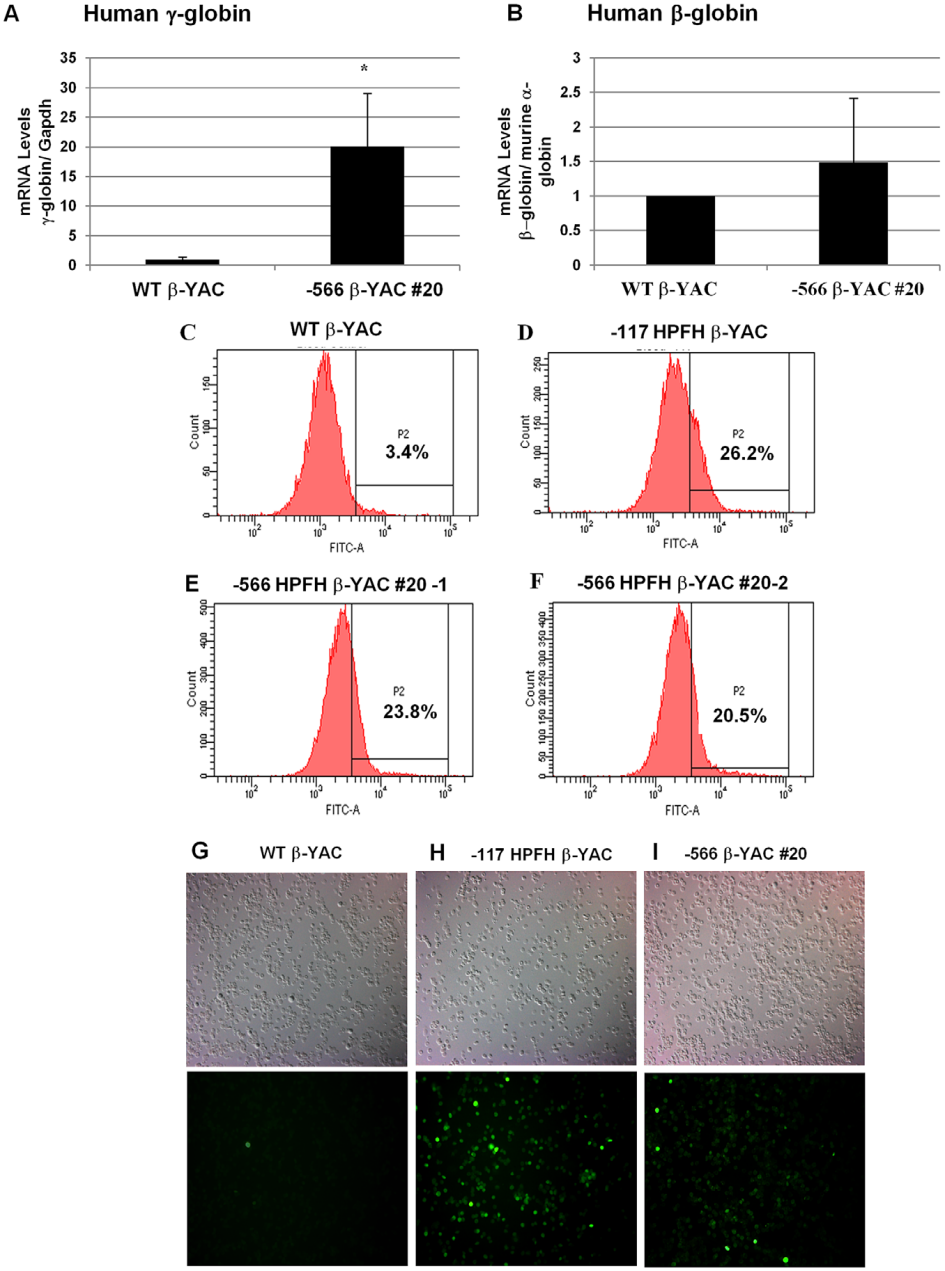
Human β-like globin expression in adult −566 ^A^γ-globin HPFH β-YAC transgenic mice. (A and B) Expression of the fetal γ- and adult β-globin was analyzed by real-time qRT-PCR and the data was normalized to mouse α-globin or Gapdh gene expression. (A) Human fetal γ-globin mRNA expression; B) Adult β-globin mRNA expression. Lines are indicated at the bottom of the plot. Results are the average of triplicate experiments from three animals of lines 20. * indicates P<0.05. (C–F) Flow cytometry analysis was performed using a mouse monoclonal anti-γ-globin antibody to determine the percentage of F cells. C) Wild-type β-YAC; D) −117 Greek HPFH β-YAC; E) −566 ^A^γ-globin HPFH β-YAC transgenic line 20 (1), F) −566 ^A^γ-globin HPFH β-YAC transgenic line 20 (2). Flow cytometry controls are identical to those shown in Figure 2A, 2B and 2G since the experiments were performed together. (G–I) Cytospins were prepared using an anti-human hemoglobin F FITC-conjugated antibody. G) Wild-type β-YAC; H) −117 Greek HPFH β-YAC; I) −566 ^A^γ-globin HPFH β-YAC transgenic line 20. Top panels, visible light images of cytospin cells; bottom panels, immunofluorescent images of same fields.

To test whether the −566 T>G point mutation reproduced a human HPFH phenotype and maintained γ-globin expression in the adult YAC transgenic mice, human β-like globin gene expression was measured by qRT-PCR in blood from F_2_ or F_3_ generation adult mice. Mouse α-globin and Gapdh served as internal controls to quantitate human β-like globin transgene expression levels. All values were normalized to these internal controls and corrected for transgene and endogenous gene copy number. Overall, the average of line 20 animals showed a 20-fold increase of γ-globin expression (P<0.05; Figure 6A) and a 1.5-fold increase of β-globin expression, but this increase was not statistically significant (Figure 6B). The variance of both γ-globin and β-globin gene expression observed among different animals from the same lines and between lines suggests that position effect variegation is operative in −566 ^A^γ-globin HPFH. However, these results clearly demonstrate that γ-globin gene expression is increased during adult definitive erythropoiesis when the −566 HPFH mutation is present. The increase is small compared to the −117 G>A ^A^γ-globin Greek HPFH, in which γ-globin transcription is induced 300-fold (unpublished data) [21], [22].

We also determined the ratio of human γ-globin protein chains to total human β-like globin protein chains (γ-globin/(γ-globin+β-globin) by reversed-phase high-performance liquid chromatography (RP-HPLC) in adult blood hemolysates from −566 ^A^γ-globin HPFH β-YAC line 20 mice compared to wild type β-YAC and the −117 Greek HPFH β-YAC transgenic mice (Table 1). The −566 ^A^γ-globin HPFH mice showed a small, but significant increase in γ-globin chain expression (7.5%) compared to wild-type β-YAC mice (5.1%), but less than that measured in −117 Greek HPFH mice (9.5%). These data corroborate the qRT-PCR data.

**Table 1:** RP-HPLC determination of γ-globin chain expression as a percentage of total human β-like globin chain expression in −566 ^A^γ-globin HPFH β-YAC transgenic mice.

β-YAC transgenic line	γ-globin/(γ-globin+β-globin)×100± SD
Wild-type	5.1+0.5
−117 ^A^ã-globin Greek HPFH	9.5+1.4
−566 ^A^ã-globin HPFH	7.5+1.5

Increased levels of γ-globin expression (F cells) were also demonstrated by flow cytometry analysis (Figure 6C–6F). The −566 ^A^γ-globin HPFH β-YAC mice showed a 23.8% and 20.5% increase of F cells (Figure 6E and 6F) compared to a wild-type β-YAC transgenic control (3.4% F cells; Figure 6C) and the positive control, the previously characterized −117 Greek HPFH β-YAC mice (26.2% F cells; Figure 6D). Immunostaining of −566 ^A^γ-globin HPFH β-YAC line 20 peripheral blood cytospins demonstrated a heterocellular distribution of F cells in this line (Figure 6I), compared to a pancellular distribution in −117 Greek HPFH β-YAC mice (Figure 6H); [21], [22]. Although only one representative microscope field is shown in each panel of Figure 6G–I, the number of positively stained cells was approximately 6-fold higher compared to wild-type β-YAC transgenic mice (Figure 6G). The modest increase of γ-globin expression associated with the −566 HPFH mutation should be therapeutic for sickle cell patients [23], [24].

### Disruption of GATA-1 mediated silencing by the −566 ^A^γ-globin HPFH mutation

**Figure 7:**
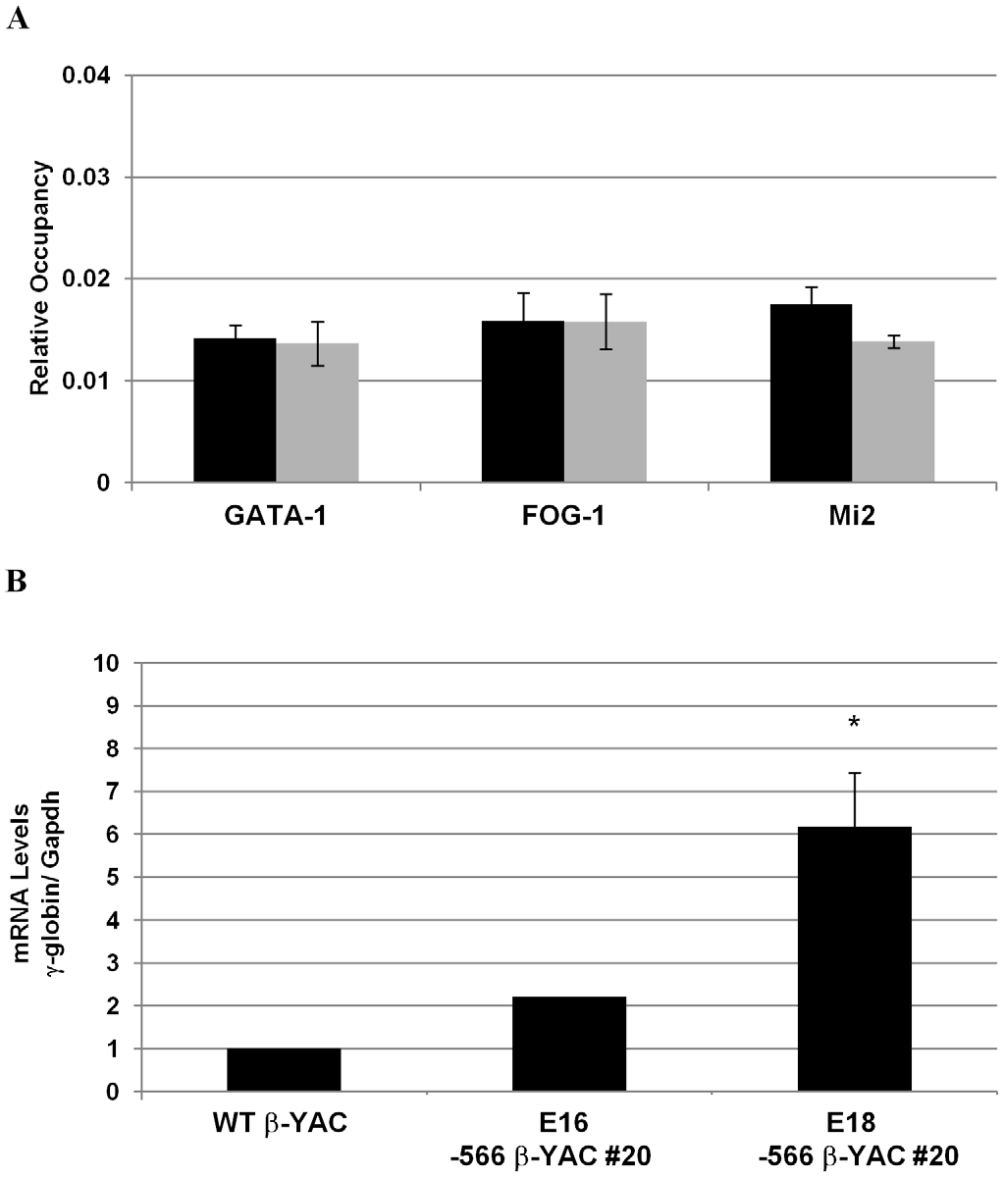
Disruption of the γ-globin silencing in the −566 ^A^γ-globin HPFH β-YAC transgenic mice. ChIP analysis of the −566 ^A^γ-globin GATA site and determination of human fetal γ-globin mRNA expression in fetal liver samples from post-conception days E16 and E18–566 ^A^γ-globin HPFH β-YAC line 20 transgenic mice. A) Relative occupancy of the −566 region by GATA-1, FOG-1, and Mi2 proteins (gray bars) is shown in comparison to the IgG control samples (black bars). ChIP was performed as described in the legend to Figure 4. B) Human fetal γ-globin mRNA expression.

To validate our hypothesis that the −566 ^A^γ-globin HPFH mutation reactivates γ-globin gene expression during adult erythropoiesis by preventing the recruitment of the GATA-1/FOG-1/Mi2 repressor complex, ChIP experiments were carried out on day E18 fetal liver samples from our −566 ^A^γ-globin HPFH β-YAC transgenic line 20. Matched samples from wild-type β-YAC mice were employed as a control, where we previously demonstrated recruitment of the GATA-1/FOG-1/Mi2 repressor complex at this developmental stage [1]. These proteins were not recruited to the −566 GATA silencer region in −566 ^A^γ-globin HPFH β-YAC transgenic mice in contrast to wild-type β-YAC transgenic mice (Figure 7A). A 6-fold average increase of γ-globin transcription was observed in the E18 blood samples from two −566 ^A^γ-globin HPFH β-YAC transgenic animals (Figure 7B). However, no significant increase was detected in E16 blood samples from three 566 ^A^γ-globin HPFH β-YAC animals. Thus, the −566 HPFH mutation prevents recruitment of the GATA-1-mediated repressor complex and reactivates γ-globin gene expression.

## Discussion

**Figure 8:**
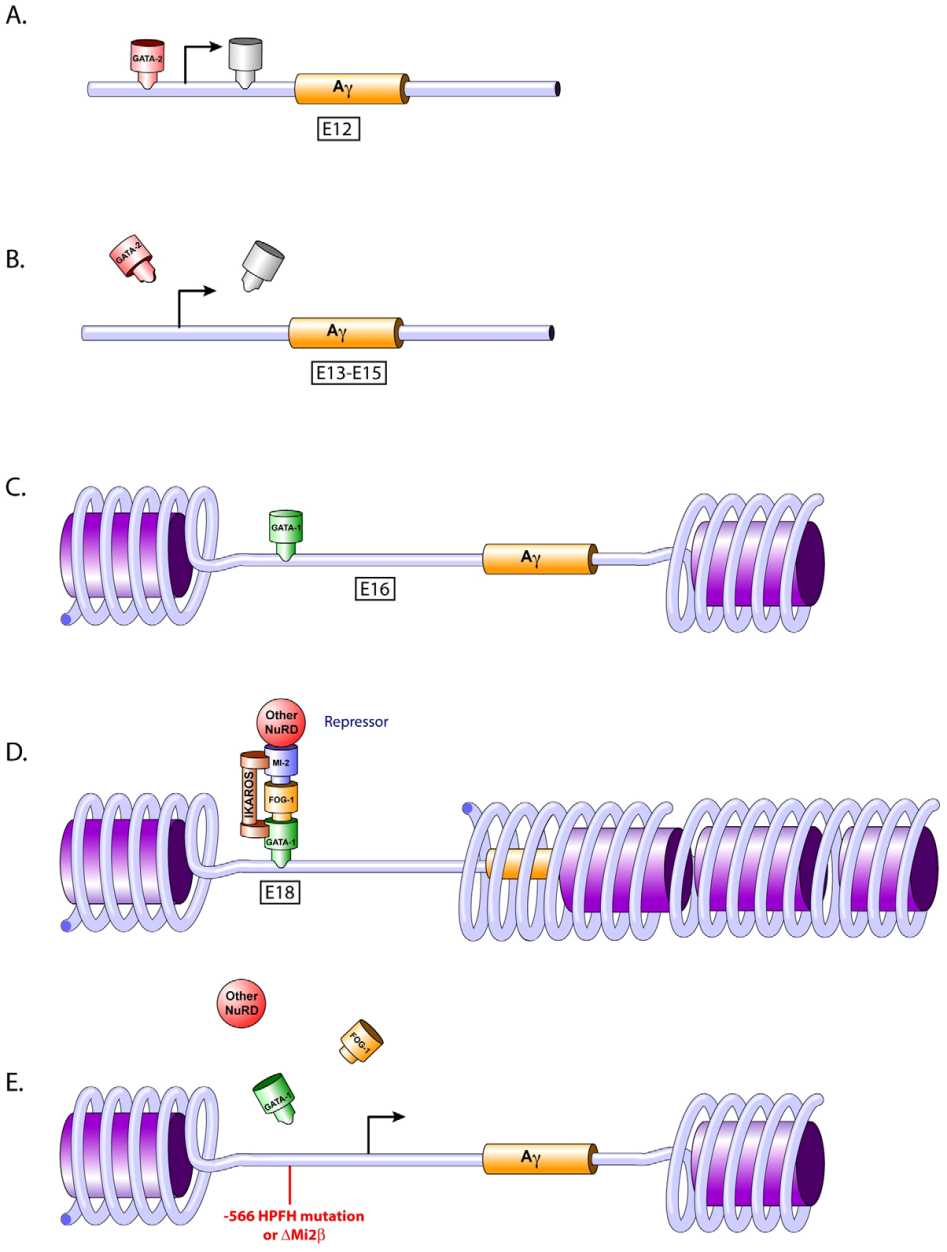
Temporal repression model. A) GATA-2 occupies the −566 ^A^γ-globin GATA gene silencer at day E12. B) Loss of transcriptional activators. The loss of GATA-2 and other transcriptional activator occupancy at the proximal promoter of the ^A^γ-globin gene at days E13 to E15 is the initial step in the silencing cascade. C) GATA-1 occupies the −566 ^A^γ-globin GATA gene silencer at day E16. A post-translational modification of GATA-1 or other determinants might play a role in this occupancy, dictating silencing versus activation by GATA-1. D) Formation of the GATA-1 repressor complex. GATA-1 recruits FOG-1 and Mi2β (and presumably other NuRD components) to the −566 ^A^γ-globin GATA silencer motif. Ikaros also may be recruited to this silencer region at a nearby Ikaros binding site where it interacts with GATA-1 and Mi2β [29]. E) Disruption of the −566 GATA site γ-globin gene repressor. γ-globin gene expression is reactivated in adult definitive erythropoiesis by preventing the recruitment of theGATA-1-FOG-1-Mi2 complex, either by the presence of the −566 ^A^γ-globin HPFH mutation or by knocking down the Mi2 subunit of the repressor complex.

Our studies provide evidence that a temporal mechanism of γ-globin gene silencing is operative at the −566 ^A^γ-globin GATA motif. GATA-1 is recruited first, at day E16, followed by the recruitment of FOG-1 and Mi2 at day E17, indicating that assembly of the GATA-1-FOG-1-Mi2 repressor complex occurs sequentially over a 24 hour period. The binding of the GATA-1 repressor complex might change the “transcription-ready” state to a more permanently silenced state by altering the chromatin into a heterochromatic state, preventing γ-globin gene transcription (temporal repression model; Figure 8).

Our data also demonstrate that the −566 GATA motif is occupied by GATA-2 early in fetal definitive erythropoiesis (day E12), followed by a change to GATA-1 occupancy at day E16, suggesting that GATA factor occupancy switching may play a role in the silencing of γ-globin expression. GATA-2 is crucial for the maintenance and proliferation of immature hematopoietic progenitors, whereas GATA-1 is essential for the survival of erythroid progenitors and for the terminal differentiation of erythroid cells [3]. Changes in global gene expression patterns during hemoglobin switching are accompanied by changes in the expression of GATA-2 and GATA-1 (GATA switching), which in part coordinates cellular maturation [3], [18]. These changes in GATA factor occupancy, combined with changes in the transcriptional factor milieu as maturation proceeds, may contribute to transcriptional repression and negative chromatin remodeling.

As human erythroid development proceeds, the proper β-like globin genes are activated or repressed, giving rise to the different hemoglobin chains expressed throughout development. Fetal hemoglobin (γ-globin) is silenced shortly after birth, and the adult hemoglobins (β- and δ-globin) are activated reciprocally. However, the γ-globin genes remain in a “transcription-ready” state, since they can be reactivated following inducing treatments such as hydroxyurea or 5-azacytidine, or by naturally occurring HPFH mutations. It is possible that the loss of GATA-2 occupancy after day E12 at the −566 ^A^γ-globin GATA site (Figure 8A) results in the simultaneous loss of transcriptional co-activators associated with GATA-2, dictating the initial event in the onset of γ-globin silencing (Figure 8A–8B). Thus, the change in GATA occupancy, from GATA-2 during early fetal definitive erythropoiesis to GATA-1 at late fetal definitive erythropoiesis observed at this site may be orchestrated by an alteration in the nearby chromatin, post-translational modification of proteins and/or changes in the transcription co-factors available in the neighborhood (Figure 8A–8C).

The demonstration of co-localization of GATA-1, FOG-1, and Mi2 by ChIP does not prove interaction between those proteins. Since we are analyzing a small region in the more distal promoter region of the ^A^γ-globin gene, it is possible that these proteins are associated with other complexes in the neighborhood, but still detected by ChIP due to the cross-linking step and size of the fragments after sonication. Hence, we do not exclude the hypothesis that other transcription factors and cofactors are recruited to nearby sites and contribute additively to silencing. Factors such as BCL11A, the orphan nuclear receptors TR2 and TR4, NF-3/COUP-TFII and Ikaros have been associated with γ-globin silencing [25]–[28]. More recently, Ikaros was shown to interact with GATA-1, since a lack of Ikaros reduced GATA-1 binding at the γ-globin promoter and delayed γ-globin gene silencing [29]. This study demonstrated that Ikaros functioned in the silencing of γ-globin by recruiting a repressor complex containing GATA-1, FOG-1, Mi2 and HDAC1.

Overall, the data presented in this study provide clear evidence of the involvement of GATA-1 and Mi2 in silencing γ-globin gene expression. In a recent study, Miccio and Blobel [10] used mutant mice expressing an altered FOG-1 that abrogated NuRD binding. The authors demonstrated that the FOG-1/NuRD interaction is dispensable for silencing γ-globin expression, but is required for FOG-1-dependent activation of human adult globin expression [10]. These data do not discriminate whether these proteins directly interact to form a mega-complex, with repressive and activator protein partners, or if a sub-population of the proteins interacts to form a distinct repressor complex and another sub-population interacts to form a distinct activator complex. A deficiency of Ikaros reduced GATA-1 binding at the ^A^γ-globin promoter, enhanced chromosomal proximity between the LCR and the ^A^γ-globin promoter and delayed γ-globin silencing. An Ikaros-related consensus binding sequence is found at the −566 position of the ^A^γ-globin gene [29], thus it is provocative to suggest that Mi2 associates with Ikaros and GATA-1 to form a fetal γ-globin repressor complex that also contains FOG-1 (Figure 8D). However, GATA-1-FOG-1 may interact with a different NuRD component, such as MTA1, and perhaps other NuRD subunits, to form an adult β-globin activator complex [10]. A significant reduction of adult-type human and murine β-like globin gene expression was observed in the bone marrow of adult β-YAC transgenic mice when the FOG-1/NuRD interaction was disrupted, suggesting that NuRD is required for FOG-1-dependent activation of adult globin gene expression [9], [10]. Bowen et al. suggested that the Mi2/NuRD complex is, in fact, a set of distinct complexes with similar biochemical properties [11]. The existence of different NuRD complex sub-types could explain the distinct roles and functions of the NuRD complex in globin regulation. One sub-type complex might be associated with activation of the adult β-globin gene and another sub-type, with shared, but also unique subunits, might be associated with repression of γ-globin gene expression. Finally, Gnanapragasam et al. demonstrated that transient knockdown of p66α and Mi2β induced γ-globin expression by 6- and 8-fold, respectively, in CID-dependent β-YAC bone marrow cells [30], which supports our data showing that Mi2 is required for γ-globin silencing.

Finally, our studies also show that maintenance of γ-globin expression observed with the −566 ^A^γ-globin HPFH point mutation resulted from the disruption of GATA-1-FOG-1-Mi2-mediated repression (Figure 8E). This finding was corroborated by the increased expression of γ-globin in the Mi2β conditional knockout lines (Figure 8E). Although the HPFH phenotype produced by the −566 ^A^γ-globin HPFH point mutation was weak, it was still at a level therapeutic for the treatment of hemoglobinopathies [23]. Heterocellular HPFH represents approximately 10% of the F cell trait population, with HbF levels between 0.8 and 5% [23]. The modest levels of γ-globin produced by the −566 ^A^γ-globin HPFH might be characteristic of a heterocellular HPFH, as demonstrated by cytospin preparations of RBCs (Figure 6G–6I). In contrast, the Mi2β conditional knockout resulted in a pancellular HPFH (Figure 2O–2P). The Mi2β knockout has a broader effect within RBCs than the *cis*-linked −566 ^A^γ-globin HPFH mutation; the loss of Mi2β may generally affect a number of γ-globin repressive mechanisms, leading to a pancellular F cell distribution, whereas the −566 mutation variably affects binding of a single γ-globin repressor complex, producing a heterocellular distribution. Data from HFPH patients bearing a mutation at the −567 ^G^γ-globin GATA motif also suggested variance in the levels of HbF caused by the point mutation. Chen et al. [2] demonstrated that the father and his 9-year-old son had moderately elevated Hb F at 10.2% and 5.9%, respectively [2]. The variance in the levels of γ-globin observed between different −566 ^A^γ-globin HPFH β-YAC transgenic animals from individual lines suggests position effect variegation (PEV) is operative. Bottardi et al. [29] demonstrated that interaction between the LCR and the ^A^γ-globin gene is reduced by binding of Ikaros to the ^A^γ-globin promoter at the time of the γ- to β-globin switch. Thus, the chromatin organization of the γ-globin promoter might be essential to maintain the long-range interaction with the LCR. The presence of the −566 point mutation may prevent the promoter from fully interacting with the LCR, blocking full engagement with the LCR necessary for complete transcriptional activation, resulting in PEV.

In conclusion, our study is the first to demonstrate the temporal assembly of a GATA-1 repressor complex in vivo. We also demonstrated that the temporal repression mechanism is disrupted by a Mi2β mutation or a HPFH mutation, alleviating the stage-specific silencing of the ^A^γ-globin gene by the GATA-1-FOG-1-Mi2 repressor complex. This mechanism potentially provides a new target for treatment of sickle cell disease and other hemoglobinopathies.

## Materials and Methods

### −566 ^A^γ-globin HPFH β-YAC construct

A 213 Kb yeast artificial chromosome carrying the human β-globin locus with the T>G ^A^γ-globin HPFH point mutation was synthesized as follows, using previously described methods [31]. Briefly, a marked ^A^γ-globin gene (^A^γ^m^) contained as a 5.4 Kb SspI fragment (GenBank file U01317 coordinates 38,683–44,077) in the yeast-integrating plasmid (YIP) pRS406 [29] was mutagenized using the Quick Change Site-Specific Mutagenesis Kit (Stratagene, La Jolla, CA). The presence of the −566 point mutation was confirmed by DNA sequencing and the mutation was introduced into the β-YAC by “pop-in”, “pop-out” homologous recombination in yeast [1]. The mark in the ^A^γ^m^-globin gene is a six-base pair deletion at +21 to +26 relative to the ^A^γ-globin translation start site allowing preliminary discrimination of the modified β-YAC from the wild-type β-YAC by restriction enzyme digestion following homologous recombination. The presence of the mutation in clones passing this test was confirmed by DNA sequence analysis of a PCR-amplified fragment encompassing the mutated region. YAC transformation, screening of positive clones, purification, and mouse transgenesis were performed as described previously [1].

### Structural analysis

Transgene and copy number structural analyses of F_2_ generation animals were performed by standard PCR, Southern blot analyses [1] and quantitative real-time PCR (qPCR) [32], [33]. Initially, structural analysis was performed by a PCR-based approach to confirm the presence of the LCR 5′HS3, ε-, γ- and β-globin genes in the −566 HPFH β-YAC transgenics (data not shown). Further structural studies were performed by Southern blot hybridization of pulsed-field gels [1]. The primer and probe sequences used were as described previously [21], [34]. The transgene copy number was established by qPCR, using the standard curve method [32], [35], comparing dilutions from the −566 HPFH β-YAC mice to samples from our wild-type β-YAC mice line 26223, which has a well characterized copy number [31], [36]. Values were normalized to the murine α-globin and Gapdh genes.

### Mi2β conditional knockout β-YAC mice

Generation of the floxed Mi2β mice and the erythroid-specific μ'LCR-β promoter (pr)-Cre recombinase transgenic mice was described previously [13], [14], [37]. These mice were crossed to obtain μ'LCR-β pr-Cre, floxed -Mi2β/Mi2β^+^ heterozygotes, which in turn were crossed with homozygous floxed Mi2β β-YAC transgenic mice to produce mice bearing an erythroid-specific Mi2β knockout and a β-YAC reporter. PCR was employed to identify the genotype of all conceptuses using the following primer sequences: wild-type or floxed Mi2β alleles: Mi-2β forward, 5′-CTCCAAGAAGAAGACGGCAGATCRT-3′; Mi-2β reverse, 5′-GTCCTTCCAAGAAGAGCAAG-3′ [37]; μ'LCR-β pr Cre transgene: Cre forward, 5′-ACGACCAAGTGACAGCAATG-3′; μ'LCR-β pr-Cre reverse, 5′-CACCGTCAGTACGTGAGATA-3′ [14]; and β-YAC: Hu ε-globin forward, 5′-TTCTTGGAAAAGGAGAATGGGAGAGAT-3′; Hu ε-globin reverse, 5′-GCAGTAAAATGCACCATGATGCCAGGC-3′.

### ChIP assay

ChIP assays were performed as described with some modifications [1]. Fetal livers from wild-type β-YAC transgenic mice at post-conception days E12–E18 were utilized. Fetal livers from −566 HPFH β-YAC transgenic mice at post-conception days E12 and E18 were employed as controls. Cross-linking was performed using a two-step dual cross-linking method [38]. Cells were incubated for 30 minutes with 1.5 mM ethylene glycol bis[succinimidylsuccinate] (EGS), followed by 1% formaldehyde (fresh paraformaldehyde) for 10 minutes at room temperature. Chromatin was sonicated to a size range between 200 and 1,000 bp. The samples were pre-cleared with species-matched normal serum. Immunoprecipitations (IPs) were carried out with anti-GATA-1, anti-GATA-2, anti-FOG-1 or anti-Mi2 specific antibodies or isotype-matched IgG (rabbit, mouse or goat) and protein G conjugated to magnetic beads (Invitrogen Dynal, AS, Oslo, Norway). The immunoprecipitate was washed, the crosslinks were reversed and the genomic DNA was purified. Recruitment of GATA-1, GATA-2, FOG-1 and Mi2 proteins was measured by real-time qPCR, using gene specific primers as described previously [1]. The antibodies used were rat anti-GATA-1 monoclonal (N6, sc-265, Santa Cruz Biotechnology, Santa Cruz, CA), rabbit anti-GATA-2 (sc-9008, Santa Cruz Biotechnology, Santa Cruz, CA), goat anti-FOG-1 polyclonal (M-20, sc-9361, Santa Cruz Biotechnology, Santa Cruz, CA) and rabbit anti-Mi2 (H-242, sc-11378, Santa Cruz Biotechnology, Santa Cruz, CA). Isotype-matched serum (Sigma Aldrich, Saint Louis, MO) and isotype-matched IgG (sc-2026, sc-2027 and sc-2028, Santa Cruz Biotechnology, Santa Cruz, CA) were used as described [1].

### Real-time quantitative PCR and RT–PCR (qPCR and qRT–PCR)

ChIP samples were analyzed in duplicate by real-time qPCR with SYBR Green dye using a MiniOpticon or CFX96 systems (Bio-Rad, Hercules, CA). To allow comparison among primer sets, input samples from each condition were diluted serially from 1∶10 to 1∶10,000 and used as standards for all PCR samples. Enrichment of protein binding to a specific DNA sequence was calculated using the standard curve method [32]. PCR primer sequences were as previously described [1] and additional primer sequences are listed in Table 2. ChIP experiments were performed using duplicate samples and each qPCR experiment was performed two to four times for each sample set. Murine GAPDH and α-globin genes were used as internal controls for the expression data. Data is shown as the mean ± the standard deviation of the mean. The Student's *t*-test was used to determine statistical significance at P<0.05 and P<0.01.

**Table 2:** Primer sets used for expression analysis.

Name	GenBank^1^	Sequence	Product size
γ-globinGI428302130	389	F - 5′ GACCGTTTTGGCAATCCATTTC 3′	164 bp
	553	R - 5′ GTATTGCTTGCAGAATAAAGCC 3′	
β-globinGI28302128	72	F - 5′ GAGAAGTCTGCCGTTACTGCC 3′	187 bp
	259	R - 5′ CCGAGCACTTTCTTGCCATGA 3′	
Hbb-y (ε^y^ mouse)GI145966796	340	F – 5′ CAAGCTACATGTGGATCCTGAGAA 3′	76 bp
	416	R – 5′ TGCCGAAGTGACTAGCCAAA 3′	
Hbb-bh1 (βh1 mouse)GI145386512	78	F – 5′ AGGCAGCTATCACAAGCATCTG 3′	111 bp
	189	R – 5′AACTTGTCAAAGAATCTCTGAGTCCAT 3′	
Hbb-b1 (β major mouse)GI218749876	344	F – 5′ GTGAGCTCCACTGTGACAAGCT 3′	62 bp
	406	R – 5′GGTGGCCCAGCACAATCACGATC 3′	
Hbb-a1 (α mouse)GI145301577	10	F – 5′ GATTCTGACAGACTCAGGAAGAAAC 3′	121 bp
	131	R – 5′ CCTTTCCAGGGCTTCAGCTCCATAT 3′	
Mi2β MouseGI118130239	2763	F – 5′ CCTCATTGTGGATGAAGCC 3′	148 bp
	2911	R – 5′GAAAGTTGAGCAGATGAAACAG 3′	
GAPDH mouseGI52139063	883	F - 5′ AGGTTGTCTCCTGCGACTTCA 3′	100 bp
	962	R - 5′ CCAGGAAATGAGCTTGACAAAG 3′	

1 GenBank coordinates are from the files listed in the name column.

Globin and Mi2β (Chd4) gene expression was measured by real-time qRT-PCR using the relative quantification, as previously described; primers sequences are listed in Table 2 [1], [33], [39].

### HbF detection by flow cytometry

Detection of HbF (F cells) and Mi2 was performed by flow cytometric analysis [40]. Briefly, mouse blood was collected from the tail vein in heparinized capillary tubes. Ten µl of whole blood was washed in PBS and fixed in 1 ml 4% fresh paraformaldehyde (Sigma Aldrich, Saint Louis, MO). The cells were centrifuged, the supernatant discarded and the pellets were resuspended in 1 ml ice-cold acetone: methanol (4∶1) for 1 minute. Cells were washed twice in ice-cold PBS/0.1% BSA and resuspended in 800 µl of PBS/0.1% BSA/0.01% Tween 20 (PBT). One µg sheep anti-human hemoglobin F FITC-conjugated antibody (A80-136F Bethyl laboratories, Montgomery, TX) or anti-Mi2 (sc-11378, Santa Cruz Biotechnology, Santa Cruz, CA) was added to 100 µl of the cell suspension and incubated for 40 minutes at room temperature. Cells were washed with 1 ml ice-cold PBS/0.1% BSA and the pellets were resuspended in 100 µl of PBT. 100 µl Alexa 488 (Invitrogen, Molecular Probes)-conjugated secondary goat anti-rabbit antibody diluted 1∶200 in PBT was added to the cell suspension as secondary antibody to the anti-Mi2 antibody, and incubated at room temperature for 20 minutes, in the dark. Cells were washed with 1 ml ice-cold PBS/0.1% BSA and the pellets were resuspended in 200 µl of PBS [41], [42]. Cells were analyzed using a BD LSRII (BD Biosciences, San Jose, CA) with an emission filter 530/30 nm (FITC/GFP). Data from 30,000 events was acquired for analysis using BD FACSDiva software (BD Biosciences, San Jose, CA).

### HbF detection by cytospin preparation

Ten µl of anti-human hemoglobin F FITC-conjugated antibody-stained cells were added to 190 µl of PBS/0.1% BSA, the liquid was placed on slides, which were spun down in a cyto-centrifuge at 700 rpm for 3 minutes. Cytospin images were acquired with a Leica DM5000 B microscope outfitted with a Leica DC500 digital camera. The Leica DC500 software runs through the Adobe Photoshop platform.

### Western blot analysis

Chemical inducer of dimerization (CID)-dependent wild-type β-YAC bone marrow cell [16] and CID-dependent floxed Mi2β Cre β-YAC bone marrow cell lysates were prepared as described [1]. Protein concentrations were measured spectrophotometrically using the Bradford assay. Forty µg cellular lysate was mixed with loading dye (50 mM Tris, pH 6.8, 100 mM DTT, 2% SDS, 0.1% bromophenol blue, 10% glycerol) and heated at 95°C for 5 minutes, followed by separation in a 10% SDS-polyacrylamide gel using Tris-glycine buffer. Western blotting was performed as previously described [1].

### Reversed-phase high-performance liquid chromatography (RP-HPLC) protocol

β-like globin protein chains were separated by RP-HPLC. Hemolysates were prepared from packed red cells by freeze-thawing in water. Briefly, half capillary tubes of blood were collected (30–40 µl) and mixed with 2 µl 50 mM EDTA. The samples were washed three times with 0.9% NaCl. The RBCs were finally resuspended in 200 µl of water, vortexed for 10 seconds, centrifuged at for 20 min at 4°C to pellet debris, and the supernatant was transferred to a fresh tube. Hemoglobin concentration was determined by adding 5 µl lysate to 995 µl Drabkin's reagent, measuring the OD_540_ and multiplying by 285.7. The sample was then diluted to 2 µg/µl in buffer A (20% acetonitrile, 0.1% TFA) and filtered through a 0.2 µm PES syringe filter. 400 µg samples were run through a Vydac large-pore C4 column (214TP54) on a Waters 600S Controller and 996 Photodiode Array Detector. Buffers used consisted of buffer A and buffer B (60% acetonitrile, 0.1% TFA). The gradient was 44 to 60% buffer B over an hour [43]. Quantitation of the human globins was performed using Empower 2 software. Seven to 12 individual samples were run for each transgenic mouse line.

### Statement of ethical approval

The animal studies were performed in strict accordance with the recommendations in the Guide for the Care and Use of Laboratory Animals of the National Institutes of Health. The protocol was approved by the Institutional Animal Care and Use Committee (IACUC) of the University of Kansas Medical Center (Protocol ID Number: 2012-2060; approved 06/20/12).
